# 滤泡T细胞淋巴瘤1例

**DOI:** 10.3760/cma.j.issn.0253-2727.2022.07.013

**Published:** 2022-07

**Authors:** 晓毅 王, 洁 于, 伟华 李, 秀芝 邓

**Affiliations:** 威海市立医院血液科，威海 264200 Department of Hematology, Weihai Municipal Hospital, Weihai 264200, China

患者，男，62岁，因“发现淋巴结肿大21个月，关节肿痛20个月，皮疹、发热1个月”于2017年10月19日入院。入院前就诊于多家医院，先后被诊断为慢性淋巴结炎并急性炎症、反应性关节炎、类风湿关节炎、类癌综合征等，予对症治疗临床症状无缓解。入院查体：面部、双手、双小腿及足部散在大小不一淡红色皮疹，部分皮肤破损、脱屑，双手指关节粗大、握拳困难。颈部、腋窝、腹股沟触及多个肿大淋巴结，大者约1.5 cm×1.0 cm，质韧，活动度差，无压痛。腹软，肝脏肋下未触及。脾脏甲乙线8 cm、甲丙线11 cm、丁戊线0 cm，质韧，表面光滑，未触及包块。血常规：WBC 6.75×10^9^/L，HGB 68 g/L、PLT 239×10^9^/L。IgG 23.5 g/L，血清免疫固定电泳IgG λ型，蛋白电泳γ球蛋白38.6％。骨髓穿刺细胞形态学示继发性贫血。骨髓细胞免疫分型：B细胞及NK细胞未见明显异常表型，可见T淋巴细胞群，具体表型CD3^dim^CD4^+^CD8^−^CD7^−^CD2^+^CD5^+^TCRγ/δ^−^，占淋巴细胞18.96％，CD7表达缺失，表型异常。骨髓细胞TCRβ、TCRγ及TCRD基因重排均阳性，IgH基因重排阴性。染色体核型：46，XY[20]。行颈部左侧淋巴结活检，可见被膜增厚，未见明显淋巴滤泡，小淋巴细胞增生，结合免疫组化，以T细胞增生为主。免疫组化：CD20（+），CD79α（+），CD3（+），CD5（+），CD10（+），BCL-2（部分+），CK-pan（−），CK5/6（−），Vimentin（+），Ki-67（30％～40％+）。病理送至天津某医院会诊示：结构破坏，小淋巴细胞广泛增生，胞核圆形或轻度不规则，浆细胞和组织细胞散在分布，可见一类大细胞，胞质丰富，胞核多不规则，核仁明显，可见双核和多核。免疫组化示大细胞CD30（+），CD15（少数+），CD45（−），PAX5（弱+），Fascin（+），MUM1（+）；原单位免疫组化示大细胞CD20（−）。背景中的小淋巴细胞：CD2（较多+），CD4（较多+），CD8（少数+），CD7（−）。诊断：（左颈部淋巴结）经典型霍奇金淋巴瘤，淋巴细胞为主型。考虑会诊结果与临床不符，标本送北京某医院淋巴瘤病理专家会诊，镜下见：淋巴结被膜纤维化、增厚，边缘窦开放，窦组织增生，淋巴滤泡不明显。血管增生明显，内皮肥胖。淋巴样细胞弥漫增生，部分似有结节状结构（图1A）。增生细胞体积中等偏小，胞质丰富、淡染，核略不规则、深染。散在大细胞，单核或双核，可见核仁，部分呈R-S细胞样（图1B），胞质丰富的组织细胞增生明显。加做免疫组化：CD4（+），CD21（FDC扩大），CXCL13（+），PAX5（大细胞+），PD1（+），CD30（散在+），BCL6（−），CD7（−），CD8（−），原位杂交：EBV-EBER（散在大细胞+）。

**图1:**
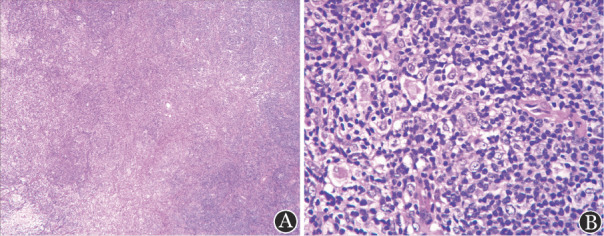
患者颈部左侧淋巴结病理 A：HE染色，低倍；B：HE染色，高倍

结合病史及原单位TCR基因重排结果诊断为（颈部左侧）淋巴结非霍奇金淋巴瘤，滤泡T细胞淋巴瘤。同时反馈病理结果至天津会诊医院病理医师处，重新阅片修正诊断为滤泡T细胞淋巴瘤。确诊后予CHOP（长春地辛+表柔比星+环磷酰胺+泼尼松）方案治疗2个周期，出现肺部真菌感染、心力衰竭，为避免蒽环类药物加重心脏损害，改为COPE（长春地辛+环磷酰胺+泼尼松+依托泊苷）方案治疗6个周期，患者临床症状缓解，但评估未达完全缓解。2018年7月患者拒绝继续化疗，亦拒绝西达本胺、来那度胺等药物治疗，仍有皮肤瘙痒、腰背疼痛及淋巴结肿痛等，调整方案予甲泼尼龙、依托泊苷、环磷酰胺、沙利度胺等控制病情，用药后症状可短期缓解，停药即加重。后因颈部淋巴结肿痛、腰椎压缩性骨折、严重带状疱疹、肺部感染等多次入院治疗，2020年10月因严重感染死亡。

